# Inferring the Dynamics of Effective Population Size Using Autosomal Genomes

**DOI:** 10.1038/srep20079

**Published:** 2016-02-01

**Authors:** Zheng Hou, Yin Luo, Zhisheng Wang, Hong-Xiang Zheng, Yi Wang, Hang Zhou, Leqin Wu, Li Jin

**Affiliations:** 1 State Key Laboratory of Genetic Engineering and Ministry of Education Key Laboratory of Contemporary Anthropology, Collaborative Innovation Center for Genetics and Development, School of Life Sciences and Institutes of Biomedical Sciences, Fudan University, Shanghai 200433, China; 2 State Key Laboratory of Surface Physics, Key Laboratory for Computational Physical Sciences (Ministry of Education), and Department of Physics, Fudan University, Shanghai, China; 3 School of Information Science and Technology, SunYat-sen University, Guangzhou, China; 4 CAS-MPG Partner Institute for Computational Biology, Shanghai Institutes for Biological Sciences, Chinese Academy of Sciences, Shanghai, China; 5 Department of Biostatistics and Computational Biology, University of Rochester Medical Center, Rochester, NY, USA

## Abstract

Next-generation sequencing technology has provided a great opportunity for inferring human demographic history by investigating changes in the effective population size (*N*_*e*_). In this report, we introduce a strategy for estimating *N*_*e*_ dynamics, allowing the exploration of large multi-locus SNP datasets. We applied this strategy to the Phase 1 Han Chinese samples from the 1000 Genomes Project. The Han Chinese population has undergone a continuous expansion since 25,000 years ago, at first slowly from about 7,300 to 9,800 (at the end of the last glacial maximum about 15,000 YBP), then more quickly to about 46,000 (at the beginning of the Neolithic about 8,000 YBP), and then even more quickly to reach a population size of about 140,000 (recently).

The dynamics of human population size provide important information for understanding the processes underlying human evolutionary history. Important events in the course of human evolution, such as the development of technological innovations and climatic changes, often led to changes in human population size, and in turn have left footprints on extant genetic polymorphism^1^,^2^. The next-generation whole-genome sequencing technology has provided a great opportunity for interrogating the human population demography^3^,^4^ through investigations of changes in effective population size (*N*_*e*_). A number of methods for estimating *N*_*e*_ from genetic data have been developed since Kingman introduced the coalescent theory^5^, and recent developments have made it possible to study changes in *N*_*e*_ using large-scale sequencing data^3^,^4^,^6^,^7^,^8^. Gronau *et al.*^3^ and Heled *et al.*^6^ estimate inter-population variation in *N*_*e*_, which provides no information on the dynamics within a population. These two methods and as well as Li *et al.*^4^’s method also have limitations in estimating the dynamics of *N*_*e*_ when dealing with whole-genome sequencing data of medium to large numbers of sampled individuals, due to either the complexity of the algorithm itself^4^ or the substantial amount of computational calculations required^3^,^6^ which is very difficult to handle using current computational resources. On the other hand, inferring the dynamics of *N*_*e*_ from the site frequency spectrum^7^,^8^ requires the assumption of independence of sites, which wastes the linkage information in the data.

East Asia, being the crossroads of human migrations and human activities, is one of the most important regions for studying both the evolution and the genetic diversity of human populations^9^. In particular, the details of the population demographic history in this region since the Last Glacial Maximum (LGM) have scarcely been investigated. Furthermore, present research is still confined to mtDNA, Y-chromosome, or only a few autosomal loci with conflicting results^10^,^11^,^12^,^13^, especially with regard to the start time and the extent of expansions.

In this report, we develop a strategy for estimating the changes in *N*_*e*_, allowing the exploration of whole-genome sequences, we call ENUMS (Estimation of *N*_*e*_ Using Multiple Segments). This strategy includes three steps: (1) identification of haplotype blocks (hereafter referred to as blocks), following the definition of Wang *et al.*^14^, (2) estimation of *N*_*e*_ dynamics through time for each block (hereafter referred to as block *N*_*e*_ dynamics) using the Bayesian Skyline Plot (BSP) method^15^, and (3) estimation of population *N*_*e*_ changes over time by taking the weighted average value of *N*_*e*_ of all blocks at each time point, which minimizes the Euclidean distance to all block *N*_*e*_ dynamics (see Methods for details). Based on the coalescence theory and by employing the standard Markov Chain Monte Carlo (MCMC) procedure, the Bayesian Skyline Plot (BSP) method^15^ can co-estimate the evolutionary rate, substitution model parameters, phylogeny and ancestral population dynamics within a single analysis directly by sampling DNA sequences. In order to reduce the noise associated with short coalescent intervals, the method allows multiple coalescent intervals to be grouped, assuming the population sizes in successive coalescent intervals are correlated. The population sizes in these grouped intervals are allowed to change linearly or remain constant. The resulting estimation of *N*_*e*_ dynamics over time is gained from the posterior sampling of the MCMC procedure, including credibility intervals that represent both phylogenetic and coalescent uncertainty. By applying the BSP method directly to each block and then integrating the information from all blocks, our ENUMS strategy not only has inherited the advantages of the BSP method, but is also able to circumvent the limitations of dealing with large samples of whole-genome sequences.

We applied this strategy to the samples of Han Chinese autosomal genomes in Phase 1 taken from the 1000 Genomes Project (1KGP)^16^ to investigate how *N*_*e*_ changed in three periods separated by two important events (i.e., the end of the LGM and introduction of agriculture). The three periods are: (1) the LGM period (25,000 YBP –15,000 YBP); (2) from the end of the LGM period to the end of the Paleolithic era (15,000 YBP – 8,000 YBP) and (3) from the beginning of the Neolithic era to recent (8,000 YBP – recent).

## Results

Genome-wide autosomal SNP (Single Nucleotide Polymorphism) sites of the 197 Han Chinese individuals (CHB & CHS) from the low coverage data set (the Phase 1 data) in the 1KPG^16^ were used in this study.

To circumvent the unspecified influence of crossovers on the BSP method^15^ which was used as part of the estimation algorithm in the following analysis, we partitioned the sequence data of each chromosome into blocks free of traces of crossover events by employing the four-gamete test (FGT) algorithm^14^ on all of the individuals under study. Given the knowledge of poor sequencing quality in the 1KGP data set, we selected 844 haplotype blocks of higher quality, 5,516,675 bp in length totally, from all the autosomal blocks (see Supplementary Results & Supplementary Table 1 for details). 332 of these blocks overlapped with at least one gene while 512 did not. Although this strategy may cause biased results since only part of each chromosome were used, it uses at least some of the linkage information by assuming no recombination within each block for inference of *N*_*e*_ (also see Methods & Discussion for details).

The BSP method^15^ was applied to individual blocks to estimate block *N*_*e*_ dynamics. Blocks with the age of the most recent common ancestor (MRCA) younger than 25,000 years were removed, leaving 801 blocks (hereafter referred to as Best Set), 315 of which overlap with at least one gene. To further simplify the description of the dynamics of each block *N*_*e*_, we denoted a vector of 26 elements corresponding to *N*_*e*_ values that were taken at a series of time points from present to 25,000 YBP with an interval of 1,000 years (also see Methods & Supplementary Figure 2).

**Figure 1:**
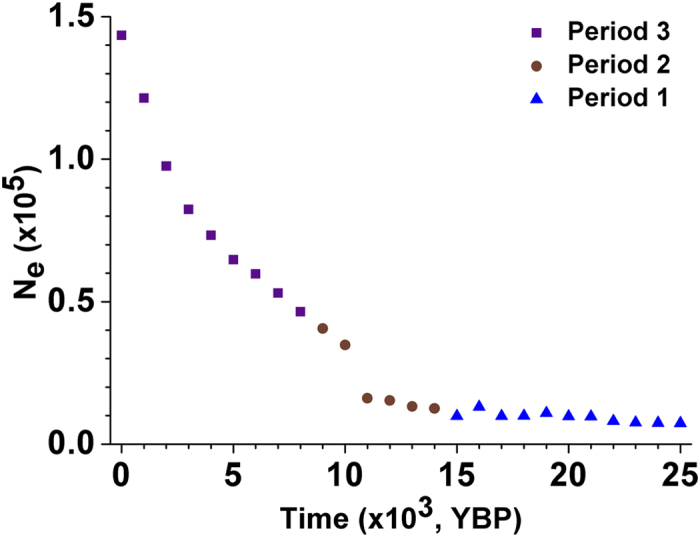
Changes of *N*_*e*_ in the Han Chinese population since 25,000 YBP. Blue triangles, brown circles and purple squares show the optimal estimation of *N*_*e*_ in Stages 1, 2 and 3 respectively.

To characterize the dynamics of the *N*_*e*_ of all blocks studied, we estimated the population *N*_*e*_ by calculating the weighted average values over the 801 blocks for every element of the aforementioned vectors (see Methods for details). The result revealed a continuous expansion from 25,000 YBP to recent, resulting in an approximately 18-fold increase in size. The initial *N*_*e*_ was ~7,300, while the recent *N*_*e*_ is ~140,000 (Fig. 1). We investigated the change of *N*_*e*_ for three time periods: (1) from 25,000 YBP to the end of the LGM (15,000 YBP), (2) from 15,000 YBP to the beginning of Neolithic (8,000 YBP), and (3) from 8,000 YBP to recent. The population increased by only 33% throughout the LGM period, with slight fluctuations. It reached ~46,000 by the end of the Paleolithic era (8,000 YBP), and then expanded to ~140,000 in size from 8,000 YBP to recent (i.e., since the invention of the agriculture^17^,^18^). When taking every block as equally weighted, a highly similar pattern of estimated population *N*_*e*_ dynamics is obtained (see Supplementary Figure 3 for details).

**Figure 2:**
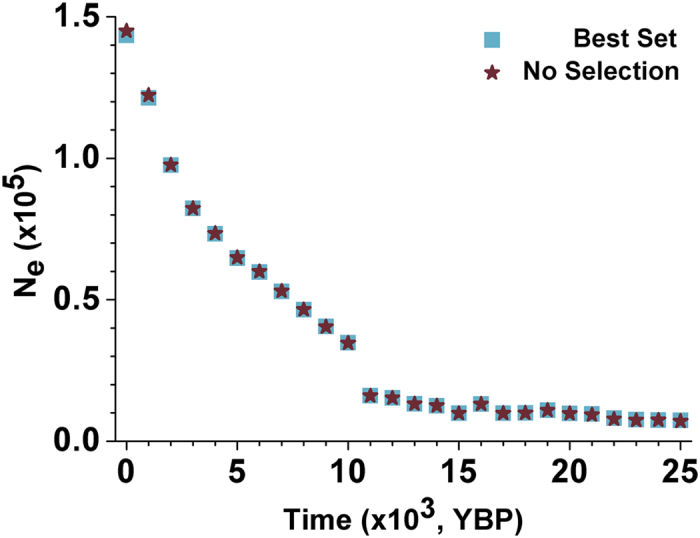
Impact of recent positive selection on the trend of estimated population *N*_*e*_ dynamics. Light blue squares represent the trend of population *N*_*e*_ estimated by all blocks in the Best Set. Brown stars represent the trend of population *N*_*e*_ estimated by the blocks in the Best Set excluding the blocks might have undergone recent positive selection.

**Figure 3:**
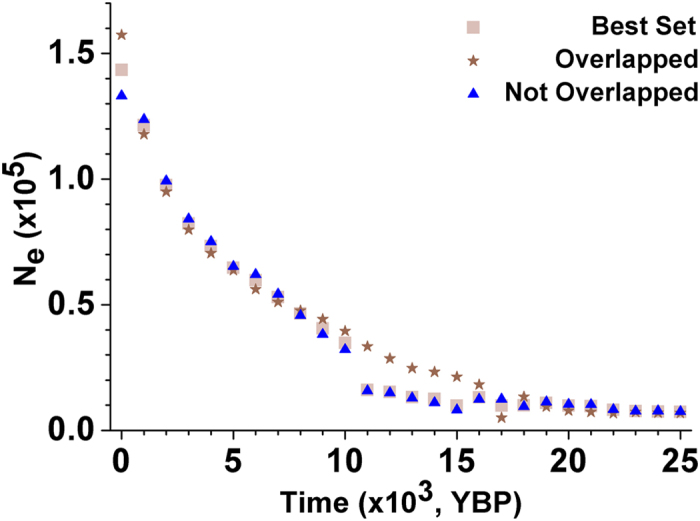
The trend of estimated population *N*_*e*_ after partitioning the Best Set into two subsets, according to whether the whole or part of the block overlaps with any of the currently known genes. Blue triangles represent the blocks that do not overlap with any genes while brown stars represent the blocks that overlap or partially overlap with one or more genes. Light squares represent the trend of population *N*_*e*_ estimated by all blocks in the Best Set.

Among all of the 25 blocks that might have undergone recent positive selection detected by the modified CMS^19^,^20^ test (see Supplementary Methods & Supplementary Figure 4 for details), seven of them overlapped with at least one gene (see Supplementary Table 2 for details) while two of them have been reported in East Asian populations (see Supplementary Table 3 for details). After removing all these 25 blocks from the Best Set, the remaining blocks show highly similar trends of population *N*_*e*_ dynamics (also see Fig. 2). We also partitioned the Best Set into two subsets according to whether the whole or part of the segment overlapped with any of the genes. Both subsets yielded highly similar *N*_*e*_ trends (Fig. 3).

Although our interest was in the Han Chinese *N*_*e*_ dynamics from present to 25,000 YBP, we also investigated the trend of *N*_*e*_ changes from 25,000 YBP to 300,000 YBP (see Supplementary Figure 5 for details). The results suggest that *N*_*e*_ increased very slowly from 50,000 YBP to 25,000 YBP (less than 3-fold) while remaining nearly the same from 300,000 YBP to 50,000 YBP.

## Discussion

In this report, we developed a strategy for estimating the changes in *N*_*e*_ from the recent to the past, allowing for the exploration of large multi-locus SNP datasets, and even whole-genome sequences. The results of the application of this new strategy on the recently released Phase 1 Han Chinese samples from the 1KGP^16^ suggest the following: a slight population expansion in East Asia during the LGM; an increase of *N*_*e*_ continuing during the post-LGM period and a population expansion escalation with the agriculture in the recent millennia.

**Figure 4:**
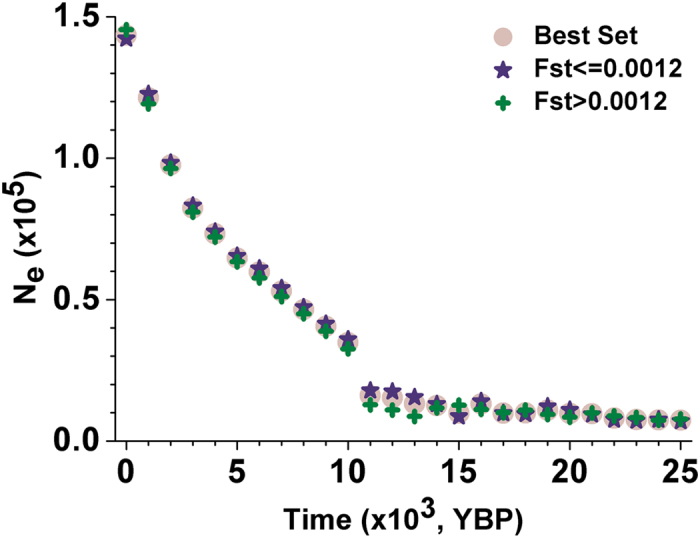
The trend of estimated population *N*_*e*_ after partitioning the Best Set into two subsets by the whole-genome average *F*_*st*_ value between CHB and CHS (0.12%). Purple stars represent the blocks with *F*_*st*_ values not more than 0.12% (512 blocks in all) while green crosses represent the blocks with *F*_*st*_ values more than 0.12% (289 blocks in all). Light circles represent the trend of population *N*_*e*_ estimated by all blocks in the Best Set.

The two sampling populations (CHB and CHS) used by this study, although collected from the extant Chinese population, constitute an effective representation of East Asia^10^,^21^,^22^. Xu *et al.*^21^ showed that both CHB and CHS are highly admixed. Furthermore, Zheng *et al.*^10^ showed that CHB and CHS have experienced similar population size changes since 25,000 YBP based on the analyses of mitochondrial genomes. Therefore, the current analyses based on CHB and CHS capture the overall picture of the demographic dynamics of human populations in East Asia. To explore how the differentiation between CHB and CHS would influence the estimation of the population *N*_*e*_ dynamics, we calculated the fixation index (*F*_*st*_)^23^,^24^ value of each block in the Best Set and the range of the resulted *F*_*st*_ values is 0 to 4.5%. We then divided the Best Set into two sub-sets according to the whole-genome average *F*_*st*_ value between CHB and CHS (0.12%)^16^. 289 blocks are with an *F*_*st*_ value more the 0.12% while 512 are not. Both sub-sets reveal highly similar patterns of population *N*_*e*_ dynamics (Fig. 4), which indicates that the influence of the population structure is very slight.

Constructing coalescent trees with recombination over a whole chromosome is an NP-hard problem when the sample size reaches tens of hundreds, therefore, it is reasonable to choose segments free of traces of crossover events in order to accomplish the estimation. However, even the FGT method, the most sensitive one for detecting currently known crossover events^14^,^25^,^26^, may fail to find all crossover events (also see Supplementary Results). Thus, how residual crossover events, although very rare (also see Supplementary Results), would influence our results is worth further investigation. We excluded any blocks shorter than 5,000 bp mainly because genealogical information may be insufficient in extremely short blocks (data not shown), but also to avoid a substantial amount of computational calculation. Generally, a 6,000 bp block consumes nearly 156 hours using one core of an XEON E5650 CPU and 10GB for hard disk of a 600,000,000-step MCMC iteration calculation and 20 GB of memory for dealing with the results generated by the MCMC iteration calculation (also see Methods for details). As a result, ~132,000 core hours of CPU and ~6TB of hard disk were consumed for all the segments in the Best Set for just one set of parameters. Meanwhile, there has not been any evidence supporting that inference is biased without short blocks^15^.

The properties of the BSP method were well studied using simulated data under different patterns of demography^15^,^27^, supporting the applicability of the method. Since the estimation of population expansion based on the ENUMS strategy is solely dependent on the BSP method (also see Methods), these simulation studies could reflect the properties and accuracy of ENUMS. Furthermore, the information on the uncertainty of the estimation of *N*_*e*_, as provided by the BSP, has been taken into consideration during the third step in ENUMS.

The times estimated by the BSP method^15^ are in the unit of mutations per site. To rescale them into the unit of years, we need to know the mutation rate per site per year. There are two approaches to estimate the mutation rate^28^ and we considered the one calculating pairwise substitution rates between closely related species as more suitable for our study (see Supplementary Discussion for details). So, we set the mutation rate as 2.5 × 10^−8^ per site per generation. However, if we choose the one counting mutation rates that occur between generations in present-day individuals, assuming the mutation rate to be 1.25 × 10^−8^ per site per generation, the results still support that the Han Chinese population has experienced a continuous expansion since 25,000 years ago (also see Supplementary Figure 6). Therefore, our results revealed autosomal expansion at least 12,000 years earlier than mtDNA expansion^10^ and 19,000 years earlier than Y-chromosomal expansion^13^ (see Supplementary Results for details).

**Figure 5:**
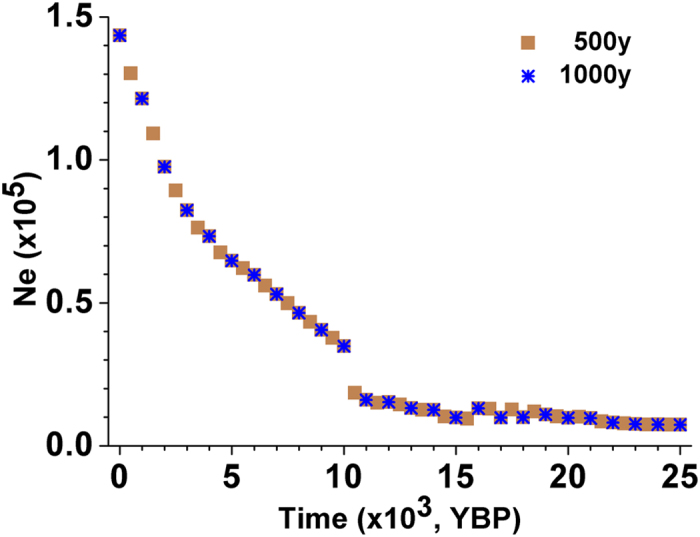
Impact of time intervals by which *N*_*e*_ values are selected for each block on the trend of estimated population *N*_*e*_ dynamics. Blue stars represent the trend of population *N*_*e*_ with a time interval of 1,000 years. Brown squares represent the trend of population *N*_*e*_ with a time interval of 500 years.

The time interval by which *N*_*e*_ values are selected for each block (also see Methods) might also have an impact on the trend of estimated population *N*_*e*_. Nonetheless, nearly the same results could be obtained after transforming the time interval from 1000 years to 500 years (Fig. 5). In addition, the quality of the dataset used in this study was not quite adequate due to the low-coverage sequencing strategy used, and thereby there may be a bias in the estimation of coalescent times as well as effective population sizes. The SNPs with ≤1% frequency were insufficient due to the poor sequencing quality^16^, especially with the inference of recent population dynamics of *N*_*e*_, since the signature of a recent population expansion will mainly be found in the singletons. A better estimation would be expected when high-quality data is available.

How recent positive selection might influence the previous estimates was investigated in two ways. Firstly, we partitioned the Best Set into two subsets according to whether the whole or part of the segment overlapped with any of the genes. Both subsets yielded highly similar *N*_*e*_ trends (Fig. 3), which also demonstrates that the amount of blocks does not influence the estimation of the population *N*_*e*_ when it is not too small. Secondly, we detected recent positive selection in each block in the Best Set by the modified CMS^19^,^20^ test (see Supplementary Methods) and 25 blocks appear to be subject to recent positive selection in total. Nearly the same trend of population *N*_*e*_ dynamics has been obtained after removing these 25 blocks from the Best Set (Fig. 2). Both the above observations suggest that recent positive selection has very limited effects on the *N*_*e*_ estimation in this study.

## Methods

### Materials

Genome-wide autosomal sequencing data of the 197 Han Chinese individuals (CHB & CHS) obtained from the low coverage data set in the 1KGP^16^ (ftp://ftp.1000genomes.ebi.ac.uk:/vol1/ftp/phase1/analysis_results/shapeit2_phased_haplotypes) were used. The OMNI^16^ dataset was used as a reference to assess the sequencing quality of each individual genome (ftp://ftp.1000genomes.ebi.ac.uk/vol1/ftp/technical/working/20111117_omni_genotypes_and_intensities/Omni25_genotypes_2123_samples.b37.vcf.gz).

### Framework of ENUMS

We have developed a strategy, denoted as ENUMS, for estimating the changes of *N*_*e*_ from the recent to the past within a population, allowing the exploration of large multi-locus SNP datasets, including whole-genome sequences. It functions by identifying haplotype blocks, estimating block *N*_*e*_ dynamics through time of each block and estimating population *N*_*e*_ changes over time by integrating information from all block *N*_*e*_ dynamics. Based on the BSP method, this strategy circumvents the limitations of large sample sizes and minimizes the Euclidean distance while integrating information from all block *N*_*e*_ dynamics.

### Estimation of block *N*_*e*_ dynamics

Generally, the dynamics of each block *N*_*e*_ are estimated by the BSP method^15^ in the program BEAST (version 1.7.0)^29^ (also see Supplementary Methods for details). No recombination is assumed within each block (also see Discussion & Supplementary Results for details). Isolation and random mating are assumed in the Han Chinese population. Each MCMC sample^15^,^30^ used in the BSP method^15^ is based on a run of 600,000,000 generations, sampled every 2,000 generations, with the first 50,000,000 generations discarded as burn-in. Specifically, the steps we took in the MCMC process were nearly 10 times longer than the ones used in other studies^10^,^31^. In order to plot the dynamics of block *N*_*e*_ with respect to time, a strict clock and a neutral mutation rate of 2.5 × 10^−8^ per generation per site^4^,^32^ and 25 years per generation are also assumed. For the convenience of analyzing all blocks together, *N*_*e*_ values were selected at a series of time points from present to 25,000 YBP with an interval of 1,000 years to describe the dynamics of each block *N*_*e*_, which can be denoted by a vector 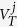, where *j* is an integer denoting the index of the block and *T* is a vector denoting the index of the time points selected, i.e., *T* = (0, 1000, …, 25000)′.

### Estimation of population *N*_*e*_ dynamics using all blocks under survey

Because estimation of *N*_*e*_ changes from any block in the Best Set may be biased by the influence of many factors such as genetic drift, positive selection, we take the vector 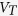 that can minimize function (1) as the estimation of the population *N*_*e*_ changes, i.e., the vector describing the population *N*_*e*_ dynamics is the one whose sum of squared Euclidean distance to all block *N*_*e*_ vectors in the Best Set is minimal (see function (2)).


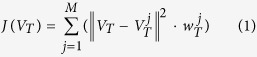



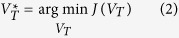


where *M* is the total number of blocks, and 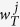 is the vector indicating the weight of 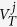 at each time point. Each element of 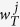 is defined as the inverse of the approximation of the standard deviation of the estimated values of *N*_*e*_ at the corresponding time point in the main result of this study.

Due to the convexity of this problem, 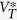 is the optimal solution of (1) if and only if


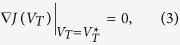


Thus the solution of (2) is


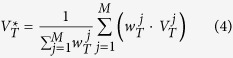


i.e., the estimation of the population *N*_*e*_.

## Additional Information

**How to cite this article**: Hou, Z. *et al.* Inferring the Dynamics of Effective Population Size Using Autosomal Genomes. *Sci. Rep.* **6**, 20079; doi: 10.1038/srep20079 (2016).

## Supplementary Material

Supplementary Information

## References

[b1] Jobling M. A., Hurles M. & Tyler-Smith C. (ed. Farrar N.) 3–12 (Taylor & Francis Routledge, 2004).

[b2] Pluzhnikov A., Di Rienzo A. & Hudson R. R. Inferences about human demography based on multilocus analyses of noncoding sequences. Genetics 161, 1209–1218 (2002). 12136023 10.1093/genetics/161.3.1209 PMC1462170

[b3] Gronau I., Hubisz M. J., Gulko B., Danko C. G. & Siepel A. Bayesian inference of ancient human demography from individual genome sequences. Nature Genet 43, 1031–1034 (2011). 21926973 10.1038/ng.937 PMC3245873

[b4] Li H. & Durbin R. Inference of human population history from individual whole-genome sequences. Nature 475, 493–496 (2011). 21753753 10.1038/nature10231 PMC3154645

[b5] Kingman J. F. C. The coalescent. Stoch Pr Ap 13, 235–248 (1982).

[b6] Heled J. & Drummond A. J. Bayesian inference of species trees from multilocus data. Mol Biol Evol 27, 570–580 (2010). 19906793 10.1093/molbev/msp274 PMC2822290

[b7] Gutenkunst R. N., Hernandez R. D., Williamson S. H. & Bustamante C. D. Inferring the joint demographic history of multiple populations from multidimensional SNP frequency data. PLoS Genet 5, e1000695 (2009). 19851460 10.1371/journal.pgen.1000695 PMC2760211

[b8] Excoffier L., Dupanloup I., Huerta-Sanchez E., Sousa V. C. & Foll M. Robust demographic inference from genomic and SNP data. PLoS Genet 9, e1003905 (2013). 24204310 10.1371/journal.pgen.1003905 PMC3812088

[b9] Zhang F., Su B., Zhang Y.-p. & Jin L. Genetic studies of human diversity in East Asia. Philos T Roy Soc B: Biol Sci 362, 987–996 (2007). 10.1098/rstb.2007.2028 PMC2435565 17317646

[b10] Zheng H-X *et al.* Major Population Expansion of East Asians Began before Neolithic Time: Evidence of mtDNA Genomes. PLoS ONE 6, e25835 (2011). 21998705 10.1371/journal.pone.0025835 PMC3188578

[b11] Xue Y. *et al.* Male demography in East Asia: a north–south contrast in human population expansion times. Genetics 172, 2431–2439 (2006). 16489223 10.1534/genetics.105.054270 PMC1456369

[b12] Aimé C. *et al.* Human genetic data reveal contrasting demographic patterns between sedentary and nomadic populations that predate the emergence of farming. Mol Biol Evol 30, 2629–2644 (2013). 24063884 10.1093/molbev/mst156

[b13] Yan S. *et al.* Y Chromosomes of 40% Chinese Descend from Three Neolithic Super-Grandfathers. PLoS ONE 9, e105691 (2014). 25170956 10.1371/journal.pone.0105691 PMC4149484

[b14] Wang N., Akey J. M., Zhang K., Chakraborty R. & Jin L. Distribution of recombination crossovers and the origin of haplotype blocks: the interplay of population history, recombination, and mutation. Am J Hum Genet 71, 1227–1234 (2002). 12384857 10.1086/344398 PMC385104

[b15] Drummond A. J., Rambaut A., Shapiro B. & Pybus O. G. Bayesian coalescent inference of past population dynamics from molecular sequences. Mol Biol Evol 22, 1185–1192 (2005). 15703244 10.1093/molbev/msi103

[b16] Genomes Project, C. *et al.* An integrated map of genetic variation from 1,092 human genomes. Nature 491, 56–65 (2012). 23128226 10.1038/nature11632 PMC3498066

[b17] Bettinger R. L., Barton L. & Morgan C. The origins of food production in north China: A different kind of agricultural revolution. Evol Anthropol 19, 9–21 (2010).

[b18] Barton L. *et al.* Agricultural origins and the isotopic identity of domestication in northern China. Proc Natl Acad Sci USA 106, 5523–5528 (2009). 19307567 10.1073/pnas.0809960106 PMC2667055

[b19] Grossman S. R. *et al.* A composite of multiple signals distinguishes causal variants in regions of positive selection. Science 327, 883–886 (2010). 20056855 10.1126/science.1183863

[b20] Grossman S. R. *et al.* Identifying recent adaptations in large-scale genomic data. Cell 152, 703–713 (2013). 23415221 10.1016/j.cell.2013.01.035 PMC3674781

[b21] Xu S. *et al.* Genomic dissection of population substructure of Han Chinese and its implication in association studies. Am J Hum Genet 85, 762–774 (2009). 19944404 10.1016/j.ajhg.2009.10.015 PMC2790582

[b22] Chu J. *et al.* Genetic relationship of populations in China. Proc Natl Acad Sci USA 95, 11763–11768 (1998). 9751739 10.1073/pnas.95.20.11763 PMC21714

[b23] Weir B. S. & Cockerham C. C. Estimating F-statistics for the analysis of population structure. Evolution 38, 1358–1370 (1984). 10.1111/j.1558-5646.1984.tb05657.x 28563791

[b24] Weir B. S. & Hill W. G. Estimating F-statistics. Annu Rev Genet 36, 721–750 (2002). 12359738 10.1146/annurev.genet.36.050802.093940

[b25] Gabriel S. B. *et al.* The structure of haplotype blocks in the human genome. Science 296, 2225–2229 (2002). 12029063 10.1126/science.1069424

[b26] Barrett J. C., Fry B., Maller J. & Daly M. Haploview: analysis and visualization of LD and haplotype maps. Bioinformatics 21, 263–265 (2005). 15297300 10.1093/bioinformatics/bth457

[b27] Ho S. Y. & Shapiro B. Skyline-plot methods for estimating demographic history from nucleotide sequences. Mol Ecol Res 11, 423–434 (2011). 10.1111/j.1755-0998.2011.02988.x 21481200

[b28] Scally A. & Durbin R. Revising the human mutation rate: implications for understanding human evolution. Nat Rev Genet 13, 745–753 (2012). 22965354 10.1038/nrg3295

[b29] Drummond A. J., Suchard M. A., Xie D. & Rambaut A. Bayesian phylogenetics with BEAUti and the BEAST 1.7. Mol Biol Evol 29, 1969–1973 (2012). 22367748 10.1093/molbev/mss075 PMC3408070

[b30] Drummond A. J., Nicholls G. K., Rodrigo A. G. & Solomon W. Estimating mutation parameters, population history and genealogy simultaneously from temporally spaced sequence data. Genetics 161, 1307–1320 (2002). 12136032 10.1093/genetics/161.3.1307 PMC1462188

[b31] Atkinson Q. D., Gray R. D. & Drummond A. J. mtDNA variation predicts population size in humans and reveals a major Southern Asian chapter in human prehistory. Mol Biol Evol 25, 468–474 (2008). 18093996 10.1093/molbev/msm277

[b32] Nachman M. W. & Crowell S. L. Estimate of the mutation rate per nucleotide in humans. Genetics 156, 297–304 (2000). 10978293 10.1093/genetics/156.1.297 PMC1461236

