# Supplementary material for: Inferring the Dynamics of Effective Population Size Using Autosomal Genomes
Source: Sci Rep. 2016 Feb 1;6:20079. doi: 10.1038/srep20079 (PMC4735516; doi:10.1038/srep20079)
Supplement: Supplementary Information [file srep20079-s1.pdf]

Inferring the Dynamics of Effective Population Size Using Autosomal Genomes

Zheng Hou[1], Yin Luo[2,§], Zhisheng Wang[3,§], Hong-Xiang Zheng[1,§], Yi Wang[1], Hang Zhou[4], Leqin Wu[5], Li Jin[1,4,*]

[1] State Key Laboratory of Genetic Engineering and Ministry of Education Key Laboratory of Contemporary Anthropology, School of Life Sciences, Fudan University, Shanghai, China.

[2] State Key Laboratory of Surface Physics, Key Laboratory for Computational Physical Sciences (Ministry of Education), and Department of Physics, Fudan University, Shanghai, China.

[3] School of Information Science and Technology, SunYat-sen University, Guangzhou, China.

[4] CAS-MPG Partner Institute for Computational Biology, Shanghai Institutes for Biological Sciences, Chinese Academy of Sciences, Shanghai, China.

[5] Department of Biostatistics and Computational Biology, University of Rochester Medical Center, Rochester, NY, USA.

[§] These authors contributed equally to this work.

[*] Request for reprints: Li Jin (email: lijin.fudan@gmail.com).

Supplementary Materials

Results

Selection of haplotype blocks

Genome-wide autosomal SNP sites of the 197 Han Chinese individuals (CHB & CHS) from the low coverage data set (the Phase 1 data) in the 1KGP[1] were used in this study (also see Supplementary Figure 1 for details). To circumvent the unspecified influence of crossovers on the BSP method[2], which was used as part of the estimation algorithm in the following analysis, the sequence data were partitioned into segments free of traces of crossover events. In particular, each autosome was partitioned into blocks by employing the FGT algorithm[3] on all the individuals under study. The blocks with average SNP density in either the left- or the right- 25%-tail were discarded to reduce the variation of mutation rate across the genome. Those blocks of 5 kb or less in size were also removed because their information is likely insufficient for estimating effective population sizes, as well as the substantial amount of computational calculation required to process this information.

Given the knowledge of the poor sequencing quality of the 1KGP data set, the sequencing quality of each fragment was evaluated using the OMNI[1] data set as a reference. Particularly, we took only the blocks with part or all of the SNP sites that are present in the OMNI[1] data into consideration. We compared each site in both datasets individually for each haplotype, to identify those that are inconsistent between the two datasets. For all the haplotypes under study, the average, minimum and maximum inconsistent rates were 8.7%, 8.4% and 9.1%, respectively, with a standard deviation of 0.1%. Thus, if the number of individuals carrying inconsistent haplotypes exceeded five in a block, then the entire block was excluded for further analyses. As a result, 844 blocks were selected overall, 332 of which overlapped with

one or more of the currently known genes while 512 did not. This set of blocks has an average SNP density of 0.54%, a bit higher than the whole-genome average SNP density (also see Supplementary Table 1 and Supplementary Figure 1).

The power of the FGT Method

The FGT method was applied to data simulated by MS[4] and msHOT[5], to calculate the power of detecting crossover events. An expansion demographic model was set. The N_e was initially 7,700 and then grew exponentially during the following 1,000 generations, finally reaching 150,000. The mutation rate used was 2.5×10^{-8} per site per generation. The sequences simulated were 2,000,000 bp in length and 394 sequences were randomly sampled. Both uniformly distributed recombination and recombination hot spot models were applied to the above demography and 500 replicates were done for each. The background crossover rate used was 1.0×10^{-8} per generation between adjacent basepairs for both situations. Three crossover hot spots were used for the hot spot model: the first one was between 100,000 bp and 102,000 bp, with the crossover rate 1.0×10^{-7} per generation between adjacent basepairs; the second one was between 700,000 bp and 702,000 bp, with the crossover rate 1.0×10^{-6} per generation between adjacent basepairs; and the third one was between 1,500,000 bp and 1,502,000 bp, with the crossover rate 1.0×10^{-5} per generation between adjacent basepairs. Both situations show that over 99% of crossover events can be detected by the FGT method.

Discussion

The time and N_e estimated by the BSP method[2] are in the unit of mutations per site. To rescale them into the unit of years, we need to know the mutation rate per site per year. There are two approaches to estimate the mutation rate[6]. One is to calculate

pairwise substitution rates between closely related species, such as humans and chimpanzees while the other is to count mutation rates that occur between generations in present-day individuals[6]. The former approach emphasizes estimating the average mutation rate over a long period of time and the estimated mutation rate is 2.5×10^{-8} per site per generation[6]. Our results based on this approach (Figure 1) revealed that autosomal expansion was about twice as long as mtDNA expansion[7] and three-times longer than Y-chromosomal expansion[8]. The estimated mutation rate based on the latter approach was generally two-times smaller than the estimated mutation rate based on the former approach[9-11]. However, if we assume the mutation rate to be 1.25×10^{-8} per site per generation[6], the results still support that the Chinese Han population has experienced a continuous expansion since 25,000 years ago (also see Supplementary Figure 6). If the mutation rate estimated by the latter approach was used, the estimated start time of the expansion in our study would be much earlier, which would lead to a much larger difference between expansion times revealed by autosomes and mtDNA under similar estimation methodology[7]. Therefore, our autosome-based study indicates an expansion that began at least 12,000 years earlier than mtDNA expansion and 19,000 years earlier than Y chromosomal expansion. A comparatively small fraction of higher mutation rates may change the long-scale average mutation rate significantly. Although the direct measurement is undoubtedly valuable, it is not enough to change the mutation rate estimated over a long time scale. Therefore, we considered the mutation rate estimated by the former approach as more suitable for population genetic based analyses, like our study.

Another challenging issue related to mutation rate is whether it is reasonable to take a constant mutation rate across blocks. It is hard to estimate a locus-specific mutation rate since it is difficult to distinguish the variance in outgroup divergence caused by variation in mutation rates and by variation in coalescent times in the population at the root of the phylogeny. In addition, we have discarded the blocks

with average SNP density in either the left or the right 25% tail to avoid blocks that may have a mutation rate that is far deviated from the neutral mutation rate (also see Supplementary Table 1). Moreover, we examined every block by mapping it to the maps of predicted bona fide CpG islands[12] (downloaded from http://epigraph.mpi-inf.mpg.de/download/CpG_islands_revisited/) and found only 13 blocks with part of their sequences overlapping with one or more of the CpG island regions. Nearly the same estimation results have been gained after removing all these 13 blocks from the Best Set (Supplementary Figure 7). Therefore, it is reasonable to take a constant mutation rate across all blocks.

Methods

Estimation of block N_e dynamics

The dynamics of each block N_e were estimated by the BSP method[2] in the program BEAST (version 1.7.0)[13]. The underlying function of N_e was specified as a piecewise linear function of N_e changes between adjacent changing points and 40 discrete linear changes were allowed in the coalescent tree. A random starting tree was used for every segment and the estimated initial root height was set to be 0.01 units. A general time-reversible (GTR) substitution model[14] without partitioning the coding regions (if any) was used. In the GTR model, a Gamma distribution is used for the site heterogeneity model and the number of Gamma categories used was 8. The priors used for the relative rates of substitutions for A↔C, A↔G, A↔T, C↔G, C↔T and G↔T all follow the distribution of Gamma [0.05, 10] with an initial value of 1. The

maximum time of a block is considered as the root height's lower 95% highest posterior density (HPD). The initial value of the population size was set to be 0.1.

Detection of recent positive selection

We evaluated whether a SNP site has been subject to recent positive selection by applying the Composite of Multiple Signals (CMS) method[15,16] with modification to the function that calculates the composite effects of the five tests[15,17-20]: To avoid the unknown bias caused by the population demography used as a priori in simulation, the genome-wide empirical *P*-value (p_i) was used to determine the significance of selection for each test[15,17-20] instead of simulation (see the function below). A block is identified as a candidate for selection if the most significant SNP site in this block has a CMS' score less than 0.01.

$$CMS' = \prod_{i=1}^{5} p_i$$

References

1 The 1000 Genomes Project Consortium. An integrated map of genetic variation from 1,092 human genomes. *Nature* **491**, 56-65 (2012).

2 Drummond, A. J., Rambaut, A., Shapiro, B. & Pybus, O. G. Bayesian coalescent inference of past population dynamics from molecular sequences. *Mol Biol Evol* **22**, 1185-1192 (2005).

3 Wang, N., Akey, J. M., Zhang, K., Chakraborty, R. & Jin, L. Distribution of recombination crossovers and the origin of haplotype blocks: the interplay of population history, recombination, and mutation. *Am J Hum Genet* **71**, 1227-1234 (2002).

4 Hudson, R. R. Generating samples under a Wright-Fisher neutral model of genetic variation. *Bioinformatics* **18**, 337-338 (2004).

5 Hellenthal, G. & Stephens, M. msHOT: modifying Hudson's ms simulator to incorporate crossover and gene conversion hotspots. *Bioinformatics* **23**, 520-521 (2007).

6 Scally, A. & Durbin, R. Revising the human mutation rate: implications for understanding human evolution. *Nat Rev Genet* **13**, 745-753 (2012).

7 Zheng H-X, Y. S., Qin Z-D, Wang Y, Tan J-Z, et al. Major Population Expansion of East Asians Began before Neolithic Time: Evidence of mtDNA Genomes. *PLoS ONE* **6**, e25835 (2011).

8 Yan, S. *et al.* Y Chromosomes of 40% Chinese Descend from Three Neolithic Super-Grandfathers. *PLoS ONE* **9**, e105691 (2014).

9 Roach, J. C. *et al.* Analysis of genetic inheritance in a family quartet by whole-genome sequencing. *Science* **328**, 636-639 (2010).
10 Abecasis, G. *et al.* A map of human genome variation from population-scale sequencing. *Nature* **467**, 1061-1073 (2010).
11 Nachman, M. W. & Crowell, S. L. Estimate of the mutation rate per nucleotide in humans. *Genetics* **156**, 297-304 (2000).
12 Bock, C., Walter, J., Paulsen, M. & Lengauer, T. CpG island mapping by epigenome prediction. *PLoS Comput Biol* **3**, e110 (2007).
13 Drummond, A. J., Suchard, M. A., Xie, D. & Rambaut, A. Bayesian phylogenetics with BEAUti and the BEAST 1.7. *Mol Biol Evol* **29**, 1969-1973 (2012).
14 Rodriguez, F., Oliver, J. L., Marin, A. & Medina, J. R. The general stochastic model of nucleotide substitution. *J Theor Biol* **142**, 485-501 (1990).
15 Grossman, S. R. *et al.* A composite of multiple signals distinguishes causal variants in regions of positive selection. *Science* **327**, 883-886 (2010).
16 Grossman, S. R. *et al.* Identifying recent adaptations in large-scale genomic data. *Cell* **152**, 703-713 (2013).
17 Sabeti, P. C. *et al.* Genome-wide detection and characterization of positive selection in human populations. *Nature* **449**, 913-918 (2007).
18 Voight, B. F., Kudaravalli, S., Wen, X. & Pritchard, J. K. A map of recent positive selection in the human genome. *PLoS Biol* **4**, e72 (2006).
19 Tang, K., Thornton, K. R. & Stoneking, M. A new approach for using genome scans to detect recent positive selection in the human genome. *PLoS Biol* **5**, e171 (2007).
20 Cockerham, C. C. & Weir, B. S. Covariances of relatives stemming from a population undergoing mixed self and random mating. *Biometrics* **40**, 157-164 (1984).

Supplementary Table S1. Attributions of blocks in the Best Set.

Statistics	Length (bp)	Average SNP Density	θ_π[1]
Minimum Value	5001	4.4×10^{-3}	4.0×10^{-5}
25% Quantile	5421	4.7×10^{-3}	4.8×10^{-4}
Median Value	6044	5.1×10^{-3}	7.3×10^{-4}
75% Quantile	7138	5.8×10^{-3}	1.0×10^{-3}
Maximum Value	22581	1.3×10^{-2}	3.7×10^{-3}
Average Value	6604.9	5.4×10^{-3}	7.9×10^{-4}
Standard Deviation	1677.8	9.7×10^{-4}	4.3×10^{-4}

Supplementary Table S2. Blocks overlapping with one or more of the currently known genes that are considered as having undergone recent positive selection in our study.

Chromosome	Length	Start Position	End Position	Gene(s) with respect to the block
8	6500	51607314	51613813	SNTG1

**4**	**5505**	**5499803**	**5505307**	**STK32B**
**6**	**6562**	**97043025**	**97049586**	**FHL5**
**12**	**5214**	**108956610**	**108961823**	**ISCU**
**12**	**6312**	**113763287**	**113769598**	**SLC24A6**
**1**	**7657**	**174337032**	**174344688**	**RABGAP1L**
**1**	**5756**	**234320816**	**234326571**	**SLC35F3**

Supplementary Table S3. Blocks considered as having undergone recent positive selection both in our study and previous studies on East Asian populations.

Chromosome	Length	Start Position	End Position	Previous Report
**1**	**5676**	**97486216**	**97491891**	**Tang et al. 2007**
**20**	**9775**	**30233584**	**30243358**	**Williamson et al. 2007**

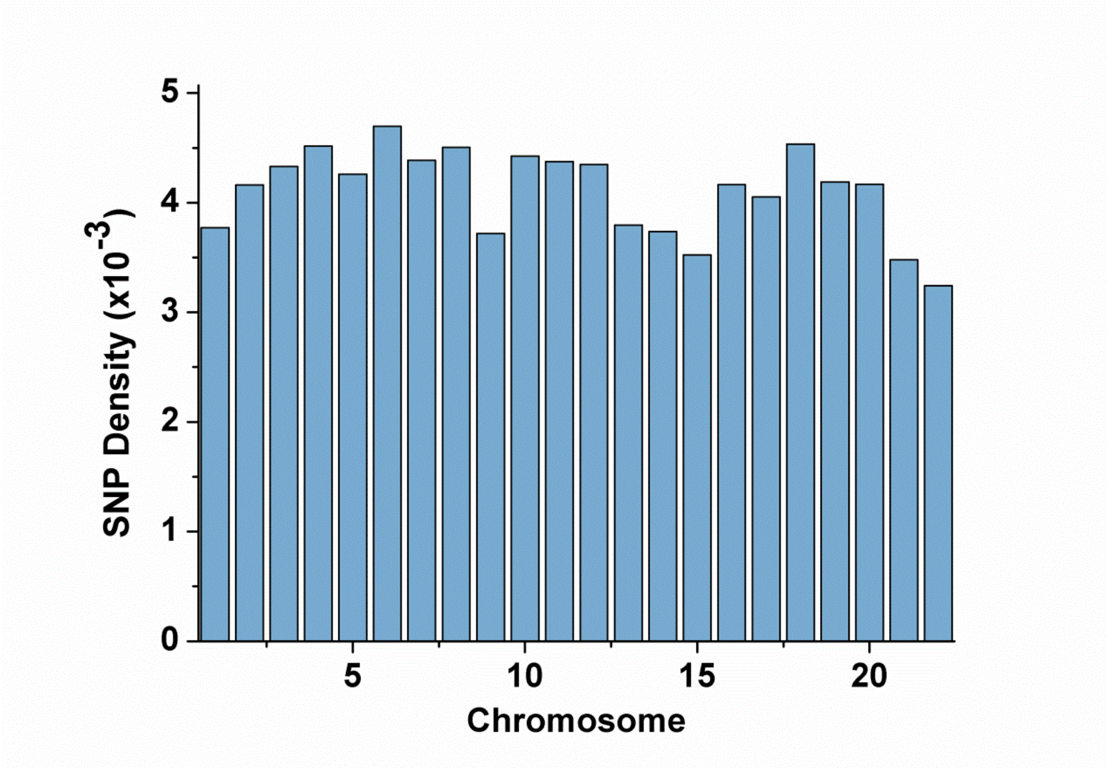

Supplementary Figure S1. The density of SNP sites by chromosome in the Phase 1 data of the 1000 Genomes Project. Each bar represents the SNP site density of a chromosome.

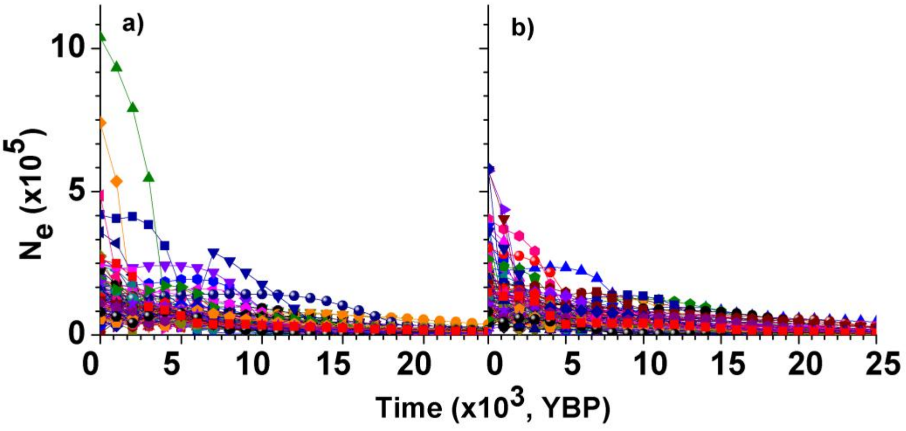

Supplementary Figure S2, (a) & (b). Each sub-figure shows the dynamics of N_e of 100 blocks randomly sampled from the Best Set. Each curve in each sub-figure describes the dynamics of the N_e of a certain block in the Best Set from 25, 000 YBP to present.

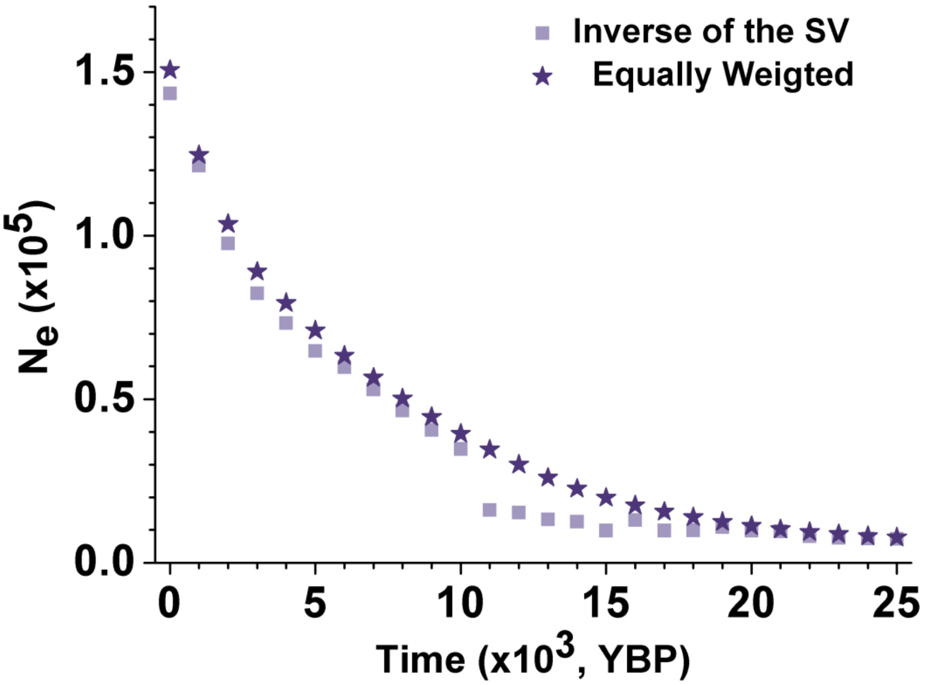

Supplementary Figure S3. Changes of N_e in the Han Chinese population since 25,000 YBP when all the blocks in the Best Set are equally weighted (purple stars). Light purple squares represent the trend of population N_e shown in Figure 1.

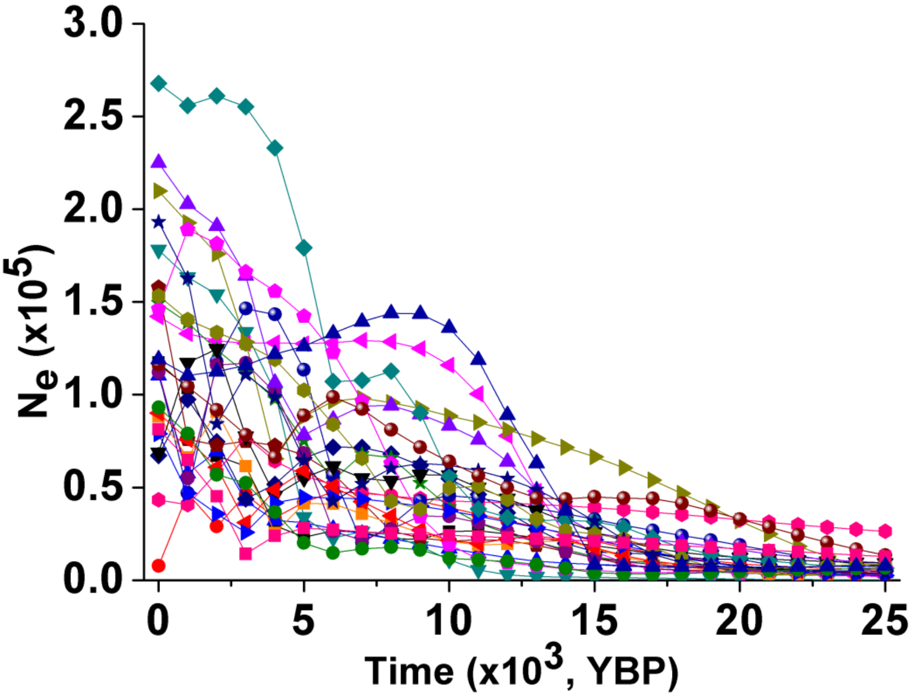

Supplementary Figure S4. Changes of N_e of all the blocks that might have undergone recent positive selection detected by the modified CMS method. Each curve describes the dynamics of the N_e of a certain block in the Best Set from 25, 000 YBP to present.

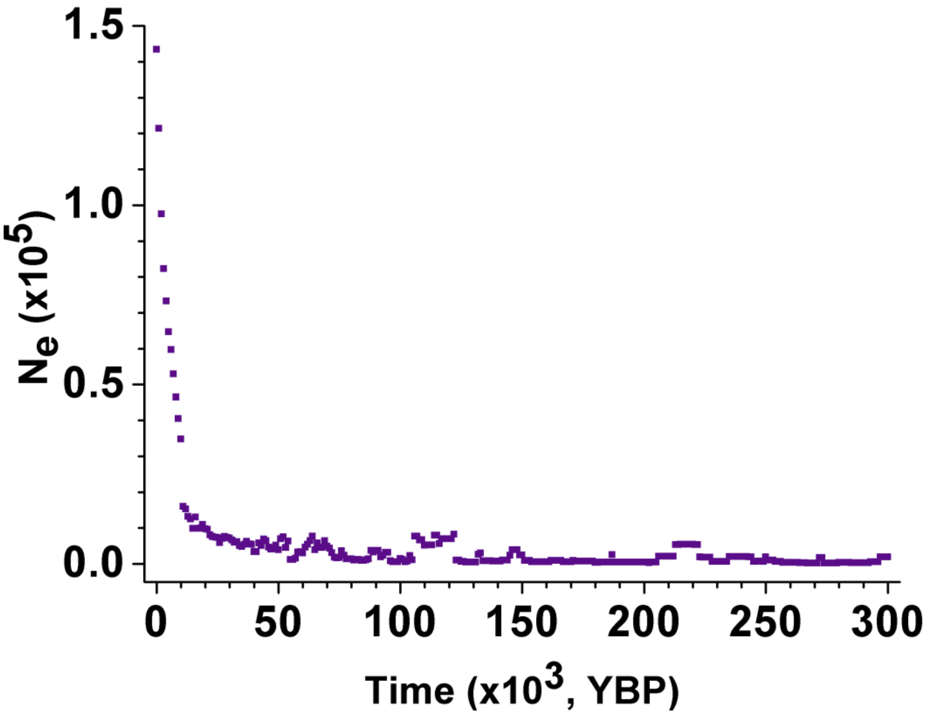

Supplementary Figure S5. The dynamics of N_e from present to 300,000 YBP. The X-axis depicts the time before present in the unit of 1,000 years; the Y-axis depicts N_e in the unit of 100,000 individuals. The purple squares represent the optimal estimation of N_e at each time point.

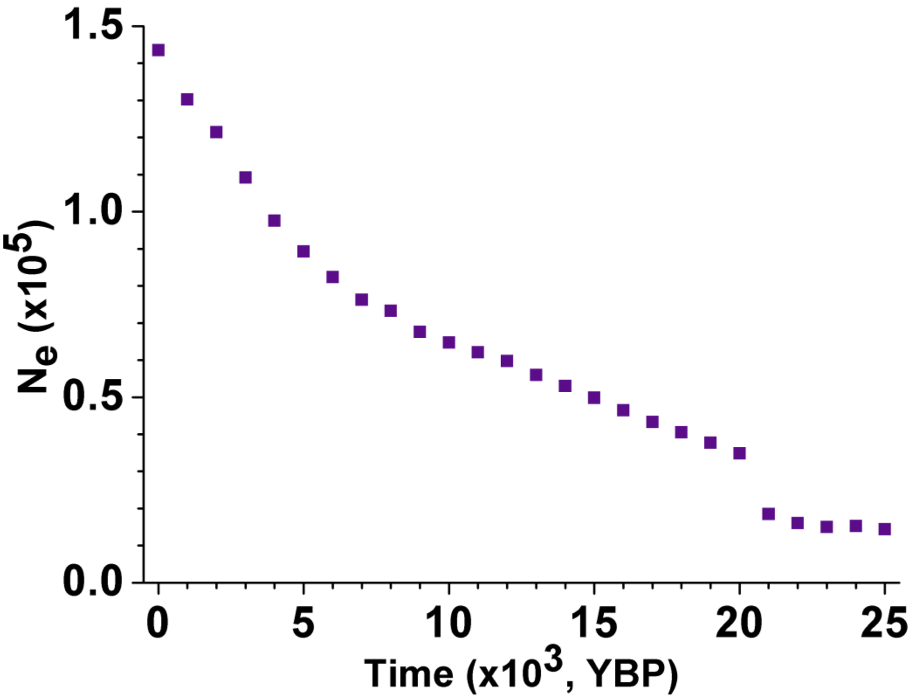

Supplementary Figure S6. Changes of N_e in the Chinese Han population since 25,000 YBP (mutation rate=1.25×10^{-8}).

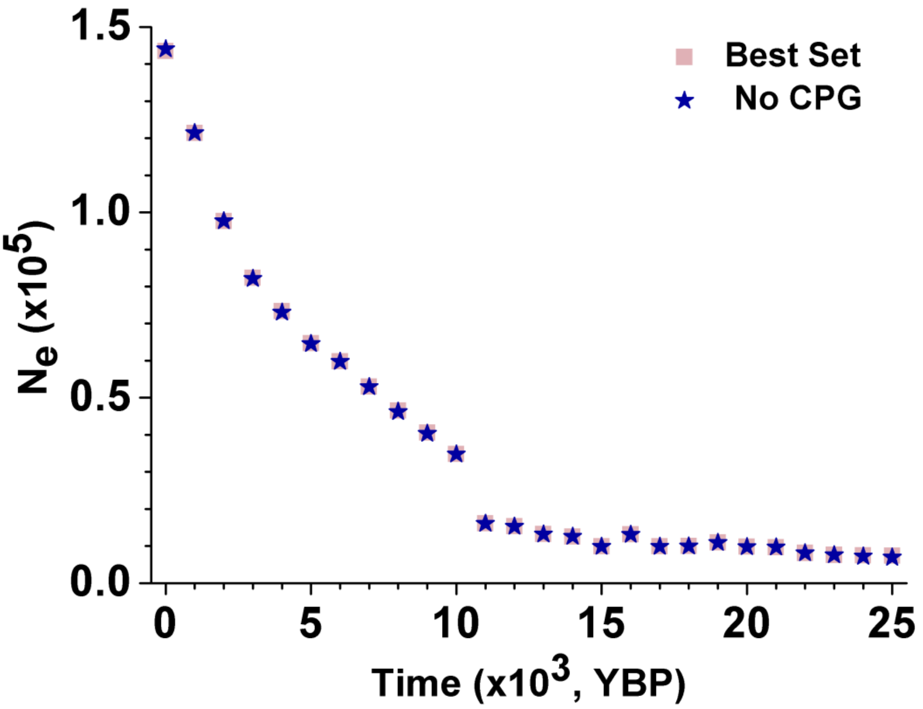

Supplementary Figure S7. Impacts of CpG Islands on the trend of estimated population N_e dynamics. Purple stars represent the trend of population N_e estimated by the blocks in the Best Set without the blocks that are partially overlapped with one or more of the CpG island regions. Light red squares represent the trend of population N_e estimated by all of the blocks in the Best Set.

References

1 Tajima, F. Evolutionary relationship of DNA sequences in finite populations. *Genetics* **123**, 437-460 (1983).
